# Draft Genome Sequence of an *ortho*-Nitrophenyl-β-d-Galactoside (ONPG)-Negative Strain of *Vibrio cholerae*, Isolated from Drakes Bay, California

**DOI:** 10.1128/genomeA.00135-16

**Published:** 2016-03-17

**Authors:** Cindy H. Wu, Chih-Ying Chen, Christina Morales, David Kiang

**Affiliations:** California Department of Public Health, Food and Drug Laboratory Branch, Richmond, California, USA

## Abstract

We present the draft whole-genome sequence of a *Vibrio cholerae* strain (Vc25-3) isolated from Drakes Bay, California. This environmental isolate has an atypical morphology and is *ortho*-nitrophenyl-β-d-galactoside (ONPG)-negative.

## GENOME ANNOUNCEMENT

Ingestion of *Vibrio cholerae* contaminated water, shellfish, and recreational water could lead to severe cholera-like gastrointestinal symptoms. *V. cholerae* has been shown to persist in the environment between outbreaks, and has been detected in broader regions than previously thought (1). Isolation of *V. cholerae* from coastal water of California and Washington has been documented (2–4). We report the isolation and whole-genome sequencing of a non-O1/non-O139/non-O141 *V. cholerae* strain (Vc25-3) from oysters grown in Drakes Bay, California. Vc25-3 has atypical white colony morphology on CHROMagar Vibrio (CHROMagar), and is *ortho*-nitrophenyl-β-d-galactoside (ONPG)-negative. In comparison, *V. cholerae* is typically ONPG-positive. The pulse-field gel electrophoresis primary and secondary patterns are KZGS12.0230 and KZGN11.0218, respectively.

Genomic DNA was extracted from overnight culture using the DNeasy blood and tissue kit (Qiagen). The genome was sequenced using Ion PGM (Thermo Fisher Scientific). SPAdes (v3.1.0) (5) was used for *de novo* assembly resulting in 156 contigs that are greater than 200 bp. The average coverage was 186×, and the contig *N*_50_ was 168,910 bp. The genome size was 4,017,337 bp, and G+C content was 47.5%. The genome was annotated through the NCBI Prokaryotic Genome Annotation Pipeline (PGAP v2.10) (http://www.ncbi.nlm.nih.gov/genome/annotation_prok/). A total of 3,796 genes were identified for the strain, and 3,333 were coding sequences (CDS).

The annotation results indicate the absence of the cholera toxin gene (*ctx*), zonula occludens toxin gene (*zot*), and *Vibrio* pathogenic island (VPI). However, sequences for accessory cholera enterotoxin (*ace*), plasmid partition gene (*par*), and toxins *HigB2* and *HipA* are identified. The *V. cholerae* ace gene causes fluid secretion (6), *par* and *HigB2* stabilize plasmids and maintain genetic stability of *V. cholerae* superintergron (7, 8), and *HipA* induces persistence and multidrug tolerance (9). A type IV pilus such as the mannose sensitive hemagglutinin (MshA, MshQ, MshO) is present. *Msh* contributes to cell survival and persistence by enhancing surface attachment and forming biofilm (5). Virulence factor proteins such as valine-glycine repeat G (*Vgr*) and *toxR* are also detected. *Vgr* is part of the type VI secretion system (T6SS), a virulence mechanism found in most Gram-negative bacteria (10).

Global climate change has led to an increase in ocean temperature. There was a 5 to 56 fold increase in *V. cholerae* numbers when water temperature increased by 2° to 5°C (11). Current sea surface temperature off the coast of California is more than 5°C above normal (http://polar.ncep.noaa.gov/sst/ophi/) due to one of the strongest El Niño phenomena predicted in the past 65 years. This could lead to a significant increase in *V. cholerae* population in the coastal waters off the Western United States, and potentially result in higher incidents of cholera-related illnesses. Despite lacking *ctx* and *zot,* some *V. cholerae* strains still have the ability to cause illness (12). Whole-genome sequencing of Vc25-3 provides valuable insights into persistence, pathogencity, and virulence of non-O1/non-O139 *V. cholerae*.

### Nucleotide sequence accession number.

The draft genome sequence of *V. cholerae* 25-3 has been deposited into GenBank under the accession number JNUX00000000.
